# Ectosialyltransferase activity: a marker for certain human haematopoietic cells.

**DOI:** 10.1038/bjc.1983.19

**Published:** 1983-01

**Authors:** W. Rossowski, B. I. Sahai Srivastava


*Br. J. Cancer* (1983), **47**, 158–160

### Short Communication

### Ectosialyltransferase Activity: A Marker for certain human haematopoietic cells

W. Rossowski & B.I. Sahai Srivastava

*Department of Experimental Therapeutics and Grace Cancer Drug Center, Roswell Park Memorial Institute, 666 Elm Street, Buffalo, N.Y. 14263.*

Ectosialyltransferase which can add sialic acid to cell surface acceptor proteins is found in many cells and its activity is elevated in transplantable and metastasizing tumour cells (Dobrossy *et al.*, 1981). Its presence on the surface of L1210 leukaemia cells has been confirmed ultrastructurally (Porter & Bernacki, 1975). Recently Maca & Hakes (1978) have reported high activity of this enzyme in two human B-cell lines as compared to two human leukaemic T-cell lines. In order to determine the value of this enzyme as a marker for certain haematopoietic cells or their differentiation, we have examined ectosialyltransferase activity in 23 immunologically characterized human cell lines of normal and various leukaemia/lymphoma origin which represent cells at different levels of maturation (Minowada *et al.*, 1981).

Twenty-three human cell lines, 3 of normal and 20 of various leukaemia/lymphoma origin were examined. The immunological characteristics of these cell lines, their origin and their differentiation stage have been described (Minowada *et al.*, 1981). All cell lines were grown in RPMI 1640 medium containing 5% heat inactivated foetal calf serum and maintained in log phase of growth by appropriate feeding. At harvest, the cell viability as determined by trypan blue exclusion test was 90–95%. Ectosialyltransferase activity was determined according to the procedure of Maca & Hakes (1978) with some modifications. Pelleted cells were washed with 50 mm N-2-hydroxy ethyl piperazine-N′, 2-ethanesulfonic acid (HEPES), pH 6.5 containing 0.9% NaCl and 10 mm $CaCl_2$. One half of the cells remained untreated whereas the other half of cells was treated with 10 units of *Vibrio cholerae* neuraminidase (Calbiochem. Behring Corp.) for 30 min at 37°C, pelleted by centrifugation (800 g) and washed with 50 mm HEPES, pH 6.5, containing 0.9% NaCl. Untreated and neuraminidase treated cells ($5 \times 10^6$) were suspended in 0.5 ml of 50 mM HEPES, pH 6.5–0.9% NaCl containing 0.1 $\mu$Ci of CMP-sialic acid [sialic—4, 5, 6, 7, 8, 9-$^{14}$C, specific activity 213 mCi mm$^{-1}$, New England Nuclear] and incubated for 60 min at 37°C. The reaction was terminated by addition of 2 ml of 1% phosphotungstic acid in 0.5 N HCl and centrifugation. The precipitated material was washed 3× with 5% trichloroacetic acid followed by absolute methanol. The pellets were solubilized in 0.5 ml of NCS solution (Amersham), mixed with 10 ml of toluene-based scintillation fluid and counted using a Packard counter.

All human cell lines examined here (Table) contained endogenous ectosialyltransferase activity as measured by incorporation of N-(acetyl-$^{14}$C) neuraminic acid from CMP-N-(acetyl-$^{14}$C) neuraminic acid to cell surface acceptor proteins. Moreover, as reported for mouse cell line L1210 (Bernacki, 1974) and human cell line Raji (Kilton & Maca, 1977), neuraminidase treatment of cells prior to labelling increased assembly of cell surface sialoproteins several times in most of the cell lines. Both endogenous ectosialyltransferase activity and that obtained after neuraminidase treatment were 2–4 times higher in B-cell lines including plasma cell line RPMI-8226 as compared to T-cell lines which is in agreement with the finding of Maca & Hakes (1978) with two T-acute lymphoblastic leukaemia (T-ALL) and two B-cell lines. No differences in above enzyme activity were found between B-cell lines of normal and malignant origin or between T-ALL cell lines representing T-blasts and T-chronic lymphocytic leukaemia (T-CLL) line SKW-3 representing more mature cells. On the other hand, pre-B cell lines NALM-1 and NALM-6 as well as non-T/non-B cell line NALM-16 expressed lower ectosialyltransferase activity, as compared to B-cell lines. In addition to B-cell lines the pre-erythroblast cell line K562 (Lozzio & Lozzio, 1979) and pre-myeloblast cell line KG-1 (Koeffler *et al.*, 1981) also had higher ectosialyltransferase activity whereas more mature myeloid/monocytoid lines ML-2, ML-3, HL-60, and U-937 had activity comparable to T-cell lines. These results indicate that ectosialyltransferase activity is lower in human T-cell lines compared to

Received 21 July 1982; accepted 28 September 1982.

0007-0920/83/010158–03 $01.00

© The Macmillan Press Ltd., 1983

**Table** Ectosialyltransferase activity of human haematopoietic cell lines

| *Cell Line* | *Origin* | *CPM* $10^{-7}$ *Cells* — *Untreated* | *Neuraminidase Treated Cells* | *Treated Cells Minus Untreated* |
|---|---|---|---|---|
| T-Cell Lines: | | | | |
| CCRF-CEM | ALL | 154±40 | 290±6 | 136 |
| CEM-A8 | ALL | 102±17 | 198 | 96 |
| RPMI-8402 | ALL | 122±15 | 329±30 | 207 |
| CCRF-$HSB_2$ | ALL | 174±25 | 274±118 | 100 |
| MOLT-4 | ALL | 152 | 377±112 | 225 |
| SKW-3 | CLL | 117±33 | 189±35 | 72 |
| B-Cell Lines: | | | | |
| RPMI-8057 | Normal | 202±24 | 892±16 | 630 |
| RPMI-1788 | Normal | 309±48 | 624±93 | 315 |
| B-89 | Normal | 144±18 | 664±75 | 520 |
| RPMI-8392 | ALL | 256±18 | 604±56 | 348 |
| HRIK | BL | 258±6 | 466±46 | 208 |
| U698M | LS | 370±33 | 1284±187 | 914 |
| JOK-1 | HCL | 378±6 | 1140±217 | 762 |
| RPMI-8226 | MM | 697±66 | 1458±213 | 761 |
| Pre-B-Cell Lines: | | | | |
| NALM-1 | CML-BP | 134±21 | 192±38 | 58 |
| NALM-6 | ALL | 126±16 | 346±78 | 220 |
| Non-T-Non B-Cell Line: | | | | |
| NALM-16 | ALL | 170±14 | 426±18 | 256 |
| Myeloid Cell Lines: | | | | |
| K-562 | CML-BP | 204±26 | 1572±777 | 1368 |
| KG-1 | AML | 570 | 1036±180 | 466 |
| ML-2 | AML | 148±20 | 338±130 | 190 |
| ML-3 | AML | 165±12 | 253±16 | 88 |
| HL-60 | APL | 135±24 | 352±28 | 217 |
| U-937 | HL (Monoblastoid) | 177±8 | 346±105 | 169 |

The above values represent mean ± standard deviation for 3 separate determinations. Values without standard deviation are means of 2 determinations. T-cell lines form non-immune rosettes with sheep red blood cells, B-cell lines have cell surface immunoglobulin, pre-B-lines have cytoplasmic immunoglobulin M and non-T/non-B line lacks T-, B-, or myeloid cell markers (Minowada *et al.*, 1981). ALL = acute lymphocytic leukaemia; CLL = chronic lymophocytic leukaemia; AML = acute myelocytic leukaemia; APL = acute promyelocytic leukaemia; CML-BP = blastic phase of chronic myelocytic leukaemia; BL = Burkitt's lymphoma; LS = lymphosarcoma; HCL = hairy cell leukaemia; MM = multiple myeloma; HL = histiocytic lymphoma.

B-cell lines and that this activity may increase along B-cell series and decrease along myeloid series with maturation. The above results most probably reflect different patterns of cell surface glyconjugates which have been proposed to be characteristic for different hematopoietic cells (Nilsson *et al.*, 1977; Krusius *et al.*, 1979; Klock *et al.*, 1981).

Supported by USPHS Grants CA-17140 and CA13038.
The authors wish to thank Dr. J. Minowada for the supply of cell lines used in this study.

**References:**

BERNACKI, R.J. (1974). Plasma membrane ectosialyltransferase activity of L1210 murine leukemia cells. *J. Cell Physiol.*, **83**, 457.

DOBROSSY, L., PAVELIC, Z.P. & BERNACKI, R.J. (1981). A correlation between cell surface sialyltransferase, sialic acid, and glycosidase activities and the implantability of B16 murine melanoma. *Cancer Res.*, **41**, 2262.

KILTON, L.J. & MACA, R.D. (1977). Nucleotide-induced inhibition of surface sialyltransferase activity on cultured Burkitt's lymphoma cells. *J. Natl Cancer Inst.*, **58**, 1479.

KLOCK, J.C., MACHER, B.A. & LEE, W.M.F. (1981). Complex carbohydrates as differentiation markers in malignant blood cells: Glycolipids in human leukemias. *Blood Cells*, **7**, 247.

KOEFFLER, H.P., BAR-ELI, M. & TERRITO, M.C. (1981). Phorbol ester effect of differentiation of human myeloid leukemia cell lines blocked at different stages of maturation. *Cancer Res.*, **41**, 919.

KRUSIUS, T., FINNE, J., ANDERSON, L.C. & GAHMBERG, C.G. (1979). Differences between the carbohydrate units of cell-surface glycoproteins of mouse B- and T-lymphocytes. *Biochem. J.*, **181**, 451.

LOZZIO, B.B. & LOZZIO, C.B. (1979). Properties and usefulness of the original K-562 human myelogenous leukemia cell line. *Leukemia Res.*, **3**, 363.

MACA, R.D. & HAKES, A.D. (1978). Differences in sialyltransferase activity and in concanavalin-A agglutination between T and B lymphoblastoid cell lines. *Biochem. Biophys. Res. Commun.*, **81**, 1124.

MINOWADA, J., KOSHIBA, H., SAGA, K. & 5 others (1981) Marker profiles of human leukaemia cell lines. *J. Cancer Res. Clin. Oncol.*, **101**, 95.

NILSSON, K., ANDERSON, L.C., GAHMBERG, C.G., & WIGZELL, H. (1977). Surface glycoprotein patterns of normal and malignant human lymphoid cells II, B. cells, B blasts, and Epstein-Barr virus (EBV)-positive and -negative B lymphoid cell lines. *Int. J. Cancer*, **20**, 708.

PORTER, C.W. & BERNACKI, R.J. (1975). Ultrastructural evidence for ectoglycosialyltransferase systems. *Nature*, **256**, 648.


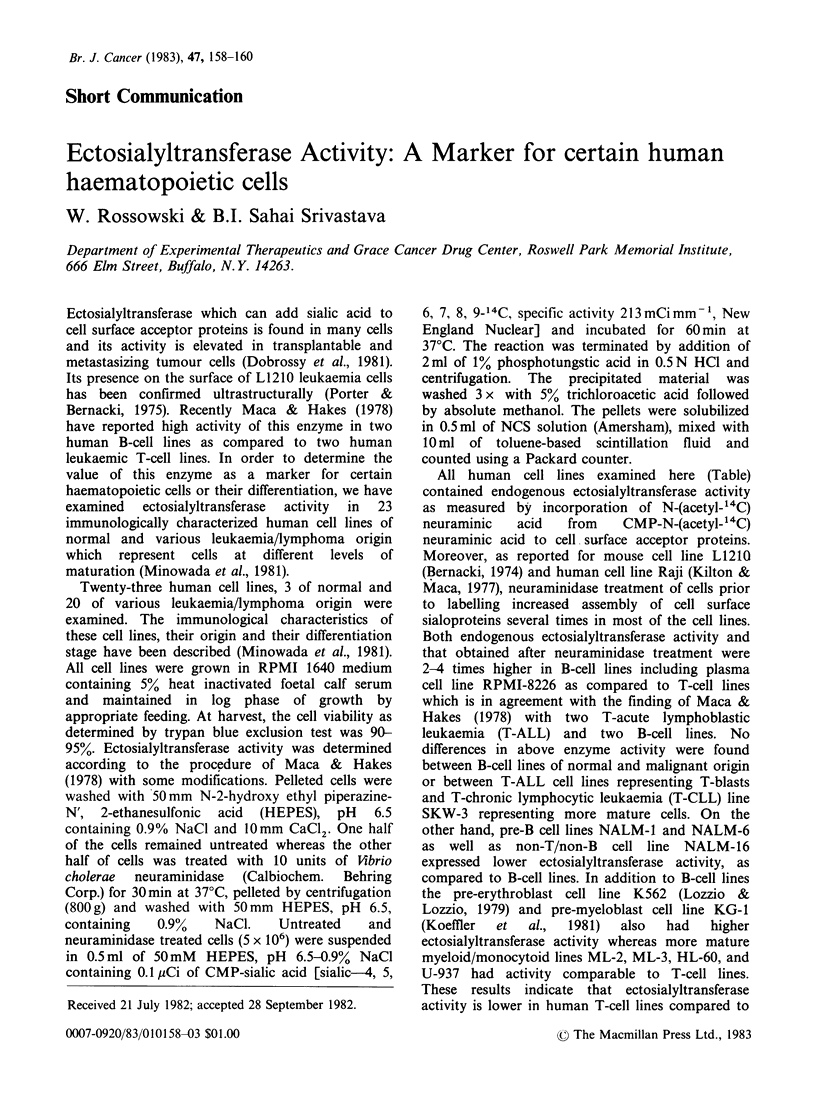

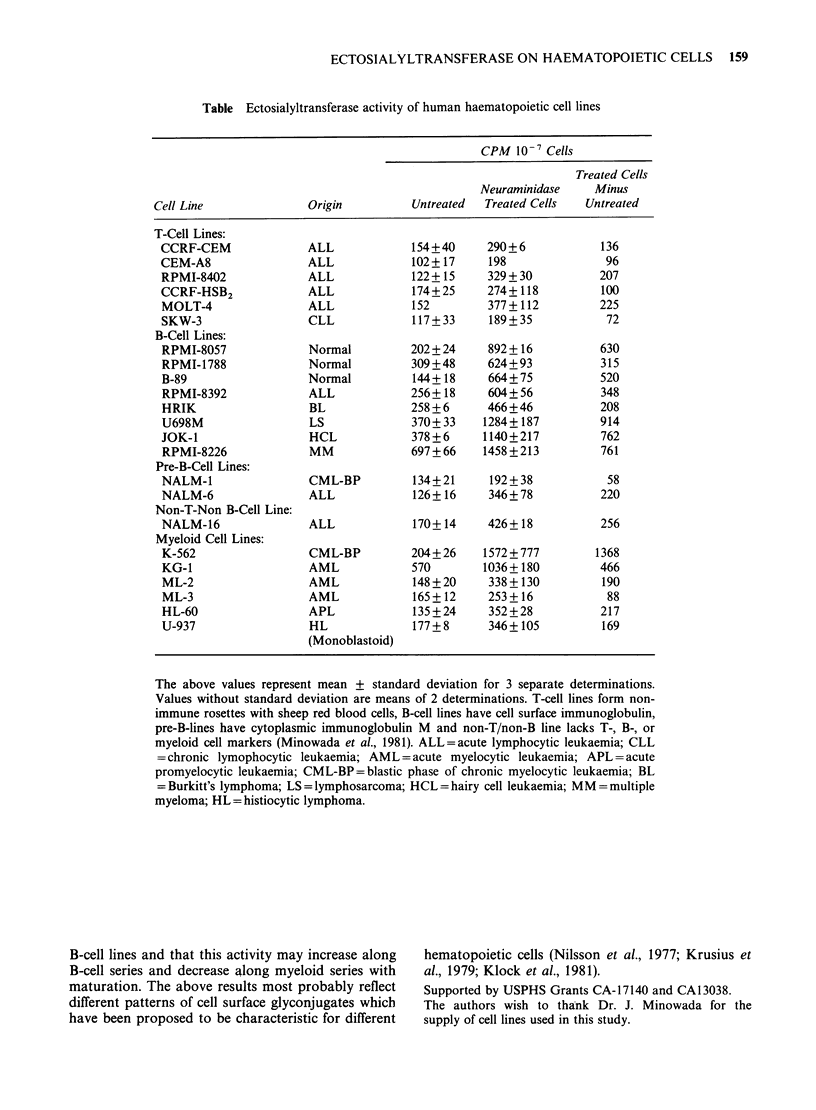

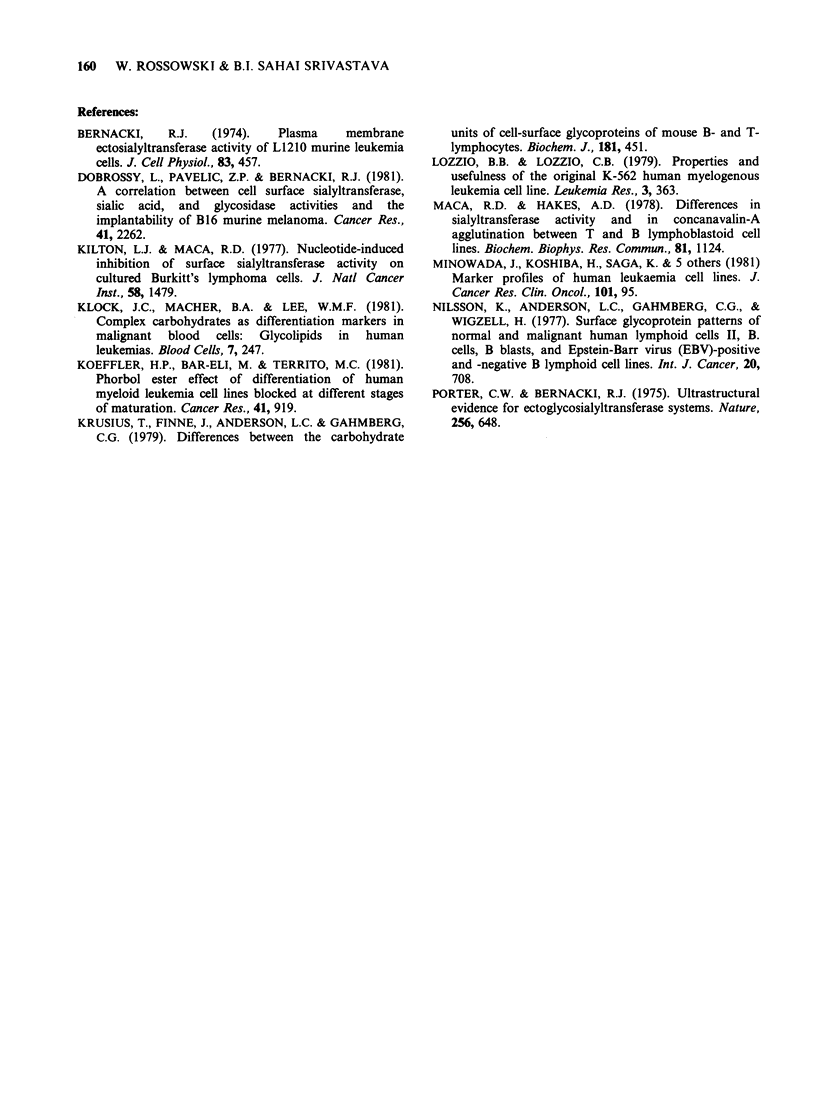


## References

[OCR_00184] Bernacki R. J. (1974). Plasma membrane ectoglycosyltransferase activity of L1210 murine leukemic cells.. J Cell Physiol.

[OCR_00189] Dobrossy L., Pavelic Z. P., Bernacki R. J. (1981). A correlation between cell surface sialyltransferase, sialic acid, and glycosidase activities and the implantability of B16 murine melanoma.. Cancer Res.

[OCR_00196] Kilton L. J., Maca R. D. (1977). Nucleotide-induced inhibition of surface sialyl transferase activity on cultured Burkitt's lymphoma cells.. J Natl Cancer Inst.

[OCR_00202] Klock J. C., Macher B. A., Lee W. M. (1981). Complex carbohydrates as differentiation markers in malignant blood cells: glycolipids in the human leukemias.. Blood Cells.

[OCR_00208] Koeffler H. P., Bar-Eli M., Territo M. C. (1981). Phorbol ester effect on differentiation of human myeloid leukemia cell lines blocked at different stages of maturation.. Cancer Res.

[OCR_00214] Krusius T., Finne J., Andersson L. C., Gahmberg C. G. (1979). Differences between the carbohydrate units of cell-surface glycoproteins of moust B- and T-lymphocytes.. Biochem J.

[OCR_00221] Lozzio B. B., Lozzio C. B. (1979). Properties and usefulness of the original K-562 human myelogenous leukemia cell line.. Leuk Res.

[OCR_00226] Maca R. D., Hakes A. D. (1978). Differences in sialyl transferase activity and in concanavalin-A agglutination between T and B lymphoblastoid cell lines.. Biochem Biophys Res Commun.

[OCR_00237] Nilsson K., Andersson L. C., Gahmberg C. G., Wigzell H. (1977). Surface glycoprotein patterns of normal and malignant human lymphoid cells. II. B cells, B blasts and Epstein-Barr virus (EBV)-positive and -negative B lymphoid cell lines.. Int J Cancer.

[OCR_00245] Porter C. W., Bernacki R. J. (1975). Ultrastructural evidence for ectoglycosyltransferase systems.. Nature.

